# Accessibility to Non-COVID Health Services in the World During the COVID-19 Pandemic: Review

**DOI:** 10.3389/fpubh.2021.760795

**Published:** 2021-12-16

**Authors:** Magdalena Tuczyńska, Maja Matthews-Kozanecka, Ewa Baum

**Affiliations:** ^1^Student Scientific Circle of Maxillofacial Orthopaedics and Orthodontics, Poznan University of Medical Sciences, Poznań, Poland; ^2^Department of Social Sciences and the Humanities, Poznan University of Medical Sciences, Poznań, Poland

**Keywords:** COVID-19, SARS-CoV2, pandemic, health services, healthcare

## Abstract

**Background:** COVID-19 pandemic caused by SARS-CoV2 has seriously impacted the global economy. Medical facilities around the world were not prepared for the enormous challenges posed by the growing number of patients each day, the shortage of personal protective equipment, and insufficient numbers of medical staff. Governments have tried to counteract the impact of the pandemic, but the measures taken have not always been sufficient to maintain access to and quality of health services at the same level as before the pandemic. The disruption of health services has resulted in more and more research reports from different parts of the world on the accessibility of health services during the COVID-19 pandemic.

**Methodology:** This review article presents 21 selected scientific studies on access to health services in different regions of the world. Articles were found in PubMed, GoogleScholar, Medline, and ScienceDirect databases, then grouped, and significant data were extracted from each article. The results were summarized in a table.

**Results:** The range of limited health services included a variety of specialties, including primary care, psychiatry, orthopedics, cardiology, neurosurgery, and more. Methods used in the studies were based on retrospective analysis or on the subjective assessment of patients in the form of a questionnaire or interview. Most authors claimed a decrease in accessibility to health services during the COVID-19 pandemic compared to the pre-pandemic period, including a decrease in planned surgeries, doctor appointments, patient admission to hospital or ER, and access to medicines. Additionally, some authors observed an increase in the mortality rate. One of the few medical services that have expanded rapidly during the pandemic was online appointments.

**Conclusions:** The COVID-19 pandemic has most certainly affected the accessibility of health services worldwide. Lessons should be learned to prevent inaccessibility to medical services, especially as experts predict another wave of COVID-19 cases.

## Introduction

The World Health Organization declared a COVID-19 pandemic on March 11, 2020 due to the novel coronavirus—SARS-CoV2. The virus belongs to the family of viruses known as coronaviruses. Coronaviruses can cause a mild respiratory infection in most immunocompetent people or can lead to more acute condition (1). The risk of more severe symptoms, including pneumonia, septic shock, sepsis, and multiple organ failure, is age-related and averages 4% per year for individuals in the 30–60 age range (2). Reduced exposure to potential infection and significant protection from SARS-CoV2 transmission is provided by surgical and N-95 masks that cover the mouth and nose. Additionally, subsequent infections are thought to have a milder course when personal protective equipment (PPE) is used (3). For personal protection during the COVID-19 pandemic, protective suits, goggles and visors, and gloves have been used in addition to masks. PPE should be made of certified materials approved by the relevant authorities. Furthermore, PPE should ensure features such as thermal comfort, breathability, and ease in the performance of procedures (4).

Access to healthcare refers to the ability to receive healthcare when needed. Economic, social, or organizational impediments can affect access to health services (5). The spread of the SARS-CoV2 virus has had a global impact on the world economy, psychosocial disparities, and access to healthcare services (6). Restrictions implemented globally included lockdowns, social distancing, and screening. In addition, the pandemic revealed, in some cases, a lack of infrastructure to protect the population and health professionals (7). Concerns that COVID-19 infection would disrupt health services grew as healthcare institutions demonstrated difficulties in responding to the impact of the pandemic (8). The challenge was to convert, in a short time, hospitals into infectious disease units for patients with COVID-19 symptoms; to supply specialized medical equipment; to supply medical personnel with PPE and provide donning and doffing areas; to organize the movement of medical personnel and patients; and to develop standard operating procedures (SOPs) (9). It was also necessary to train all medical staff in the use of PPE and in disinfection of wards to ensure work safety, and to reassure staff (10). Implementation of appropriate triage procedures throughout all health services was also required to prevent potential infection (11). The new procedures included: triage *via* telephone (to establish patients' need of treatment), symptom questionnaire, temperature check on arrival, and confirmation of the COVID-19 status (12). As far as maintaining safety in hospitals, screening for SARS-CoV2 infection has also proven to be useful (13). It is worth to mention that the triage procedure should take into account the medical need and benefit of the therapy and be based on the protocol that allows the medical staff to easily assess the status of patients (14). Another important thing during the outbreak of the pandemic is manpower reallocation planning and the need for more healthcare workers (15). Even at the beginning of the COVID-19 pandemic, there were suggestions to mobilize medical staff because of lack of them in hospitals. It was also suggested that quarantined and senior surgeons should participate in telemedicine (16). Some facilities divided their personnel for ones working in the COVID-19 zone and others working in COVID-19 free zone (17). As recommended by the Centers for Disease Control and Prevention guidelines, some hospitals have decided to reschedule non-urgent visits to ensure patient and personnel safety (18). On the other hand due to SARS-CoV2 pandemic, some patients have canceled medical appointments by themselves. Survey conducted by Hsieh et al. indicated that older age, heterosexuality, low confidence in coping with COVID-19, high general anxiety, avoiding crowded places, washing hands more often, and wearing a mask more often were significant factors for missing an appointments due to the COVID-19 pandemic (19).

The COVID-19 pandemic has caused a decrease in access to routine and even life-saving non-COVID-19 health services worldwide, which put a pressure on Ministries of Health to cut public spending or divert resources to the COVID-19 response. Low-income and middle-income countries lost from this disruptions (20). High-income countries have also faced difficulties in access to health services. Despite various sources of funding for healthcare services, access to rehabilitation care in Poland has been worsening every year. The implementation of sanitary restrictions due to the COVID-19 pandemic has significantly reduced or stopped for example rehabilitation services, though waiting times for such services has increased (21). In addition in Germany, outbreak of SARS-CoV2 led to cancellations of routine vaccination appointments, which were only rescheduled or caught up as the pandemic eased (22).

Many more studies appear in the literature on the decrease in access to health services. The aim of this study is to present the accessibility of health services during the COVID-19 pandemic in different regions of the world. We hope that this review will be a resource for professionals in various disciplines as it shows different outcomes in different periods of COVID-19 pandemic and points that many countries struggle with its outcomes.

## Methodology

The authors of the study conducted a review of scientific literature. Both first authors searched the PubMed, GoogleScholar, Medline, and ScienceDirect databases. Authors screened articles using MeSH (Medical Subject Headings) terms: “Healthcare Quality, Access and Evaluation,” “Delivery of Healthcare,” “Health Services Accessibility,” and “Healthcare Surveys.” They also added the terms: “COVID-19,” “pandemic,” and “SARS-CoV2.” The investigation included studies that met the criteria: studies on accessibility to health services worldwide during the COVID-19 pandemic, all age groups, a wide range of health services, studies based on subjective patient assessment, and studies in all languages. Studies that did not meet the criteria were excluded. These were studies focused on the accessibility to health services in the world before the pandemic only, letters to editor, and studies with insufficient data. The studies that met the inclusion criteria were listed and further reviewed. In case of bias, the second author was the decisive person. It was essential during the screening process to find research-based data from different countries and different medical specialties.

## Results

About 21 publications from the examined literature were selected for this review. From each of the included studies, the following data was extracted: author, year, country, scope of health services, type of study, outcome, additional conclusions, access to health services, age range of the study group, and comments (Supplementary Table 1). Among the 26 chosen studies, 19 were retrospective studies, four were questionnaires, one was a case study, one was a cohort study, and one was an interview. The studies discussed diverse healthcare areas, namely: mental health (*n* = 5), general health (*n* = 9), pediatrics (*n* = 2), orthopedics (*n* = 1), neurosurgery (*n* = 1), pulmonology (*n* = 1), cardiology (*n* = 1), telemedicine (*n* = 1), rheumatology (*n* = 1), oncology (*n* = 2), dentistry (*n* = 1), and maternal and child care (*n* = 1). In the majority of cases, the decrease in health service provision was related to the implementation of restrictions to increase safety and minimize the risk of infection. Lockdowns instituted by governments have restricted movement and access to medical facilities. In only one of the articles cited, it was made clear that the decrease in access to health services was linked to the increase in COVID-19 cases. From the articles cited, it also appears that the studies were conducted in age-diverse groups because SARS-CoV2 virus can infect at any age, whereas the risk of disease increases with age (23). The risk factors of infection also include male sex, chronic diseases (diabetes, hypertension, coronary artery disease, and cancer), and environmental factors (24). In addition, COVID-19 is more severe in people over the age of 60 (25). The studies included in this review involved adults including those over the age of 60, and children.

### Admissions to the Medical Facilities

During the COVID-19 pandemic, planned and urgent admissions to the medical facilities were reduced. In Germany and Italy, authors observe decreased admissions in Emergency Department (ED) among pediatric patients. Additionally in Germany, frequency of daily visits decreased for non-communicable and communicable diseases (26, 27). The reduction was also observed in Denmark, where first-time hospital admissions due to incident, oncology, and connective tissue diseases among adults substantially lowered then pre-pandemic period (28). Lockdowns have also resulted in reduced psychiatric emergency admissions with anxiety disorders as the most represented diagnosis for Spanish and Swiss patients, whereas for German patients, the greatest decrease in intellectual disability, neurotic, stress-related, and somatoform disorders, and affective disorders (29–31). In Italy, a decrease in admission to the cardiology unit was observed. Additionally, authors claimed that STEMI was the most frequent condition and patients complained less frequently of angina and more often of dyspnea on admission (32). When it comes to other services, dental admissions in Italy decline during lockdowns and the second wave of COVID-19 (33). Moreover, in Germany, during lockdowns, neurological emergency admissions of adults have declined, and out of all conditions, a decrease in admissions due to brain tumors was the highest (34). For oncology patients in the questionnaire-based study, the majority of the respondents experienced incidents of reduced access to medical services during the COVID-19 pandemic (35), and for patients with orthopedic injuries in Hong Kong, a decrease in the number of emergency visits was observed (36). Due to the transition of some facilities that were used to provide essential services to COVID-19 isolation units caused that patients with chronic illness in Kenya had difficulty getting the help they needed (37).

### Hospitalizations, Surgeries, and Medical Consultations

Hospitalizations due to COPD and asthma same as the primary diagnosis of pneumonia and influenza with pre-existing COPD or asthma decreased substantially in South Korea for patients with pulmonary disease (38). A questionnaire-based study among patients with rheumatic disease in Poland showed that the majority of the respondents experienced limitations in inpatient care, planned diagnostic hospitalization, and operating procedures (35). For patients with orthopedic injuries in Hong Kong, a decrease in the number of planned or urgent surgeries and hospitalizations was observed (36). A study carried in Australia reported that 2,085 out of 8,910 statistical areas within the Australian geography standard had a significantly high level of difficulty accessing primary health services (39). A statistically significant decrease was found in being in contact with an oncologist, family physician, and nurse among women with breast cancer in Israel (40), and for Brazilian oncology patients, a decrease in appointments for all medical specialties such as gastrointestinal/urogenital, breast, gynecology, head and neck, skin, thoracic, and other specialties was observed (41). A decrease in health service provision and utilization for antenatal, maternal, and child care was observed in a survey-based study from Bangladesh. Compared to the pre-pandemic period, fewer health facilities provided antenatal services, anthropometric measurements, prescription of and counseling on iron and folic acid (IFA), and calcium supplementation. Delivery of counseling on diet diversity, food intake, weight gain, and rest were also decreased (42). In South Africa, a retrospective study that included adults and children showed a decrease only in child health visits, and there was no change in primary care visits for adults (43). In Mexico, on the other hand, a decrease was found in sick child visits and contraceptive services by ~50%, the number of diabetes, hypertension, and antenatal care consultations by ~33.3%, and the number of deliveries attended at The Mexican Institute of Social Security by 10% (44). Authors from Romania found that the rate of continuous hospitalization decreased due to cardiovascular diseases by 46.99%, digestive diseases by 47.37%, respiratory diseases by 37.28%, and the rate of admission due to mental and behavioral disorders decreased by 55% for continuous hospitalization and ~67% for day hospitalization (45). A retrospective study in France showed a decrease in emergency psychiatric consultations during the 1st weeks of the national COVID-19 lockdown, the decrease, especially concerning anxiety disorders, mood disorders, and psychotic disorders. Total suicide attempts also declined (46). In the United Kingdom, there was a sharp reduction at lockdown in referrals to primary care mental health services, psychological therapy. This drop encompassed services accepting referrals from professionals (47). The authors who conducted a study on US patients with COVID-19 living in Florida found that lower COVID-19 cases were reported for the northwestern part of the state; however, the insufficient number of healthcare facilities and specifically ICU beds led to low access levels for the residents of these areas. In contrast to northwest Florida, there are many healthcare facilities along with more ICU beds in southern Florida. However, the high number of patients with COVID-19 in these areas led to findings of low access in these regions (48).

### Access to Medicines

A questionnaire-based study conducted in Nigeria showed that the access to essential medicines was lower during the COVID-19 pandemic than in the pre-pandemic period, which worsened the chronic conditions for patients. Additionally increase in medicine cost for acute and chronic disease was observed, so people began to seek alternative methods of treatment (49). Similar outcomes were observed in Nepal, where the supply of medicines was the worst affected area of healthcare delivery next to immunization and maternity services (50). Furthermore, in Kenya, HPV vaccination was disrupted, and BCG, pentavalent, rotavirus, and pneumococcal vaccination were declined in Mexico (37, 44). Significant declines in receipt of IFA supplements for pregnant women were observed in Bangladesh (42).

### Comparison of Access to Health Services Between Developed and Developing Countries

Both developed and developing countries have experienced a decline in the accessibility to health services due to the COVID-19 pandemic. Healthcare services that have experienced the greatest decline in developed countries included areas such as admission to the hospital for non-communicable diseases or ED, the number of planned and urgent surgeries, the number of psychiatric consultations, or psychiatric admissions to hospitals, including psychiatric admissions to the ED, whereas in developing countries, a decrease in the number of first-time medical or specialist clinic appointments was found. There has also been a substantial decline in healthcare services in these countries in the area of the provision of medicines, and compulsory vaccinations have been postponed or canceled (26–42, 44–51).

### Fields Where Increase in Health Services Was Observed

The COVID-19 pandemic period led to an increase in the delivery of services through telemedicine. A study conducted in Canada demonstrated an increase in the number of rural patients who received at least one telemedicine visit. The rate of telemedicine visits increased significantly from before to during the COVID-19 pandemic among patients with various chronic disease conditions, with the highest rates observed among patients in the mental illness subgroup, congestive heart failure, asthma, hypertension, diabetes, angina, and chronic obstructive pulmonary disease (51). An increase in access to telemedicine services was also observed in patients with rheumatic disease in Poland (35). Authors of a retrospective study from Kenya found an increase in health facility baby deliveries and measles vaccination (37). In France, the percentage of consultations for psychotic disorders increased (46), and in Denmark mortality rate ratios remained higher for infectious diseases overall (with and without COVID-19), cancer, and respiratory diseases in periods between national lockdowns (28). There was a clear and sustained increase in death among patients with psychiatric disorders in the UK at the time of lockdown, with the excess deaths occurring shortly after the rapid increase in COVID-19 infections in the region (47), same in Italy, but in patients with cardiac disease; however, after excluding patients with COVID-19, rates of in-hospital all-cause death did not differ (32).

## Discussion

The outbreak of the COVID-19 pandemic has undoubtedly had a significant impact on access to health services worldwide. Studies have shown that there has been a decline in the number of submissions to hospitals during COVID-19 pandemic compared to the pre-pandemic period for both emergency and planned surgeries. Furthermore, referrals to primary care and the number of first medical appointments and consultations have been decreased. On the other hand, online visits during COVID-19 pandemic have been increased. The questionnaire-based studies revealed that patients experienced reduced access to healthcare services, had a specialist clinic appointment canceled, or did not receive medical care when needed. In addition, in low-income countries, there has been a problem with the accessibility of medicines and difficulties with transportation to the hospital during lockdown, and the need for private services was often beyond financial resources. Some authors stated as additional conclusion an increase in the mortality rate. Others pointed significant decrease in psychiatric emergency admissions. As the results showed regardless of the country, states struggled to provide health services.

### COVID-19 and Mental Health

Regarding anxiety and depressive symptoms reported due to lockdown, the survey conducted by Fullana et al. showed that these symptoms were observed in 65% of respondents. The authors mentioned a healthy/balanced diet, following a routine, limitation on reading COVID-19-related news, pursuing hobbies, and spending time outdoors as the best methods to minimize the symptoms (52). Despite the enormous challenge that the COVID-19 pandemic created for society, it also brought an important experience for both patients and physicians. Uniting in a crisis has promoted mutual help and understanding between professionals in different fields and has led into even more conscientious patient care. Regardless of fear for themselves and their loved ones, patients and physicians demonstrated strength in the most critical moments (53).

### Telemedicine During COVID-19 Pandemic

During the COVID-19 pandemic, it has been particularly important to discover alternative methods of providing health services. Among the many solutions in the wake of social distancing, telemedicine, defined as the use of technology to provide clinical care at a distance, has proven to be a good method (54). The concept of telemedicine is broad and ranges from online consultations for symptomatic patients and those who need advice related to general illness *via* telephone or video conferencing using devices that monitor vital signs (55). Telemedicine can provide instant access to specialist physicians who are often not physically available at a given time. A limitation of telemedicine is access to other healthcare professionals, for example, nurses and medical assistants who contribute to patient care (56). The use of telemedicine, especially during the COVID-19 pandemic, has been associated with health infrastructure issues. The main drawbacks of telemedicine appeared as the breakdown of the relationship between patients and the medical staff/health professionals, the quality of information, and organizational and bureaucratic difficulties. Technological advances and the acquisition of experience by healthcare professionals can minimize the risk of these drawbacks (57). The challenge is the use of telemedicine in developing countries, where despite encouragement for consultations and the use of virtual platforms to engage with patients, caregivers, and healthcare workers, most not economically well-off patients had challenges using these platforms (37). Telemedicine requires funding and appropriate guidelines (58), especially for the elderly and those living in rural areas (59). In Uganda, due to the COVID-19 pandemic, telemedicine has expanded, patients were able to consult with healthcare providers *via* mobile phone applications, and mobile laboratory services involving providers picking specimen/samples from the location (home) of a client were noted. Despite the fact that telehealth has enabled bridging the gap of continued access to healthcare services during the COVID-19 pandemic in Uganda, there were some noted challenges such as geographical limitations, content only availed in English, or need of extra funding (60). On the other hand in some developing countries, for example, Poland, studies showed an increase in telemedical consultations. Moreover, the overall level of satisfaction from telemedicine was evaluated as more than good (35). The effort of the Indian government for expanding the use of telemedicine in the face of the COVID-19 pandemic along with private sector initiatives offers promise for mitigating the dire limitations of healthcare. As it expands, medical college hospitals and large government hospitals in the states would provide teleconsultation services to primary healthcare centers (61). Telemedicine has found particular use for patients with psychiatric disorders. Some patients have expressed fear of becoming infected with coronavirus while in the ED, which compounded their feelings of distress. To reduce the risk to both patients and staff, many consultations and assessments could be conducted *via* telephone visits (62).

### Ethics During COVID-19 Pandemic

Based on ethics, it is fundamental to ensure that patients non-infected by COVID-19 get access to healthcare (20), and when it comes to patients with severe illness, even during the pandemic, ICU patient admission should be routinely applied method, but due to shortage of ICU beds, the criteria for the patient selection are modified and patients are referred as palliative, which shakes ethical convictions (63). Furthermore, it is important to note that the outbreak of the COVID-19 pandemic sparked a worldwide discussion regarding ethics, if only because the process of vaccine development, testing, and evaluation progressed very quickly. Questions have also been raised as to whether the outbreak of a pandemic justifies controlled human infection studies and what justification there is for the professional obligation to continue work under circumstances of increased risk (64). In addition, attention was drawn to the lowered standard of reviewing published research. Research papers were published very quickly, and after a time, some of them were withdrawn due to doubts about the data used (65). Withdrawn research papers pose a risk due to insufficient marking, indicating withdrawal or access to copies on social media and pirated open access sites (66).

## Conclusion

This review points out that regardless of the country during the COVID-19 pandemic, everyone had to struggle with the outcomes for health services. We all should learn lessons for the future to prevent a decline in health services, especially as public health experts predict another COVID-19 wave. Governments should implement appropriate statutes to deal with pandemics in the future. As the above review has shown, the COVID-19 pandemic has affected the accessibility of health services worldwide. Compared to the pre-pandemic period, there has been a decrease in hospital admissions and ED visits, referrals to primary care, planned and urgent surgeries, and psychiatric consultations. Appointments at specialist clinics were canceled by facilities or by patients themselves. Availability of vaccinations and medications has also declined, especially in developing countries. There was an increase in the number of patients admitted to cardiac units, and the mortality rate also rose. As a result of the COVID-19 pandemic, hospitals and clinics have been providing telemedicine medical services, resulting in increased access to these services.

## Author Contributions

MT and MM-K designed the concept, helped in the literature search, and wrote the manuscript. EB was the decision-maker in case of bias and edited and revised the manuscript. All authors contributed to the article and approved the submitted version.

## Conflict of Interest

The authors declare that the research was conducted in the absence of any commercial or financial relationships that could be construed as a potential conflict of interest.

## Publisher's Note

All claims expressed in this article are solely those of the authors and do not necessarily represent those of their affiliated organizations, or those of the publisher, the editors and the reviewers. Any product that may be evaluated in this article, or claim that may be made by its manufacturer, is not guaranteed or endorsed by the publisher.
