# Supplementary material for: Accessibility to Non-COVID Health Services in the World During the COVID-19 Pandemic: Review
Source: Front Public Health. 2021 Dec 16;9:760795. doi: 10.3389/fpubh.2021.760795 (PMC8716399; doi:10.3389/fpubh.2021.760795)
Supplement: Supplementary file 1 [file Table_1.docx]

**Table 1. An overview of studies’ typical outcomes regarding accessibility to health services across the world during the COVID-19 pandemic.**

| **Author, year** | **Country** | **Scope of health services** | **Type of the study** | **Outcome** | **Additional conclusions** | **Access to health services** | | **Age range** | | **Comments** | |
| --- | --- | --- | --- | --- | --- | --- | --- | --- | --- | --- | --- |
| Dopfer et al., 2020 (23) | Germany | Emergency Healthcare/ Pediatrics | Retrospective | A 63.8% decrease in the number of patients admitted | Significant decrease (58%) in daily visits for noncommunicable diseases | Decreased compared to pre-pandemic period | | Mean age: 7.1 | | Comparison of number in emergency department visits between 1.01.2019 to 19.04.2019 and 1.01.2020 to 19.04.2020 | |
| Chen et al., 2020 (24) | UK | Mental Health | Retrospective | During lockdown, a rapid decline in referrals to primary care for psychological therapy | There was a gradual decrease in emergency calls and a slight immediate decrease in triage. Increase in mortality. | Decreased compared to pre-pandemic period | | - | | - | |
| Awucha et al., 2020 (25) | Nigeria | General Health - access to medicines | Questionnaire | Most common disease: malaria and hypertension - patients before the pandemic used traditional treatments; during the pandemic: increase in alternative treatments. Problem with medication accessibility. | Increase in the cost of medications for chronic diseases and acute conditions. | Decreased compared to pre-pandemic period (medications for chronic diseases) | | 0-61+ | | Subjective evaluation of respondents to the questionnaire | |
| Wong & Cheung, 2020  (26) | Hong-Kong | Orthopedics | Cohort study | Decrease in: number of planned surgeries by 73,5% ,emergency operations by 21.2%, hospitalizations by 41,2% and clinical outpatient visits by 29.3% | Patients did not endure longer wait times for emergency operations and accident and emergency consultations | Decreased compared to pre-pandemic period | | ≤18 - >65 | | Comparison from 43 hospitals and 122 outpatient clinics in the  region | |
| Pignon et al., 2020 (27) | France | Mental Health | Retrospective | A 54.8% decrease in the number of psychiatric consultations | Decrease in suicide attempts | Decreased compared to pre-pandemic period | | - | | Comparison of the 4 weeks of 2020 with the 4 weeks of 2019 | |
| Krenzlin et al., 2020 (28) | Germany | Neurosurgery | Retrospective cohort study | Decrease in admissions to ER by 44%; for tumors by 61%; for brain injuries 53% | Admissions from other hospitals to the ER without significant change | Decreased compared to pre-pandemic period | | 16-96 | | Coparison between the period 16.03.2020 to 19.04.2020 and the same period in 2019 and 2018 | |
| Lakhani, 2020 (29) | Australia (Melbourne) | General Health | Case study | Only 2085 out of 8900 areas were classified as having significantly high levels of access to healthcare services | A comparison of travel times to palliative care and hospitals showed that the time to travel during the pandemic was 9.64 minutes. | No comparison with pre-pandemic period | | 65+ | | - | |
| Nabhen et al., 2020 (30) | Brazil | Oncology | Retrospective | Decrease in number of first-time medical appointments by 42% during COVID-19 | Decrease in appointments for medical specialties: gastrointestinal/urogenital by 40%; breast by 41%; gynecology by 55%; head and neck by 17%; skin by 36%; thoracic by 26%; other 43% | Decreased compared to pre-pandemic period | | - | | Comparison of appointments in 2020 (20 March-30 June) to those in 2019 | |
| Shinan-Altman et al., 2020 (31) | Israel | Oncology | Questionnaire | A statistically significant decreases found in: contact with an oncologist, contact with a family physician, being in contact with a nurse before the COVID-19 outbreak and during. | About half of the participants reported being in isolation since the COVID-19 outbreak;35% had additional diseases (41.4% hypertension, 17.1% diabetes, 15.7% coronary heart disease, 25.7% other diseases); 31% reported cancelling a health services appointment due to the COVID-19 outbreak. | Decreased compared to pre-pandemic period | | 23-86 | |  | |
| Huh et al., 2021 (32) | South Korea | Pulmonology | Retrospective | Reduction in admissions for pneumonia, COPD and asthma | Admissions for diabetic ketoacidosis, intracranial hemorrhage and myocardial infarction did not change significantly | Decreased compared to pre-pandemic period | | - | | Comparison of pandemic admissions to average patient admissions from January 2016 - January 2019. | |
| Bodilsen et al., 2021 (33) | Denmark | General Health | Cohort Study | Hospital admissions decreased during the pandemic in the first and second lockdown | Increased mortality rate | Decreased compared to pre-pandemic period | | 0 - 81 | | - | |
| Ghorbanzadeh et al., 2021 (34) | USA (Florida) | General Health - patients with COVID 19 | Retrospective | Low accessibility to healthcare services | In the southern part of the state a large number of ICU beds but also a large number of patients with Covid -19, comparing to other parts of the state. | No comparison with pre-pandemic period | | 65 | | - | |
| Singh et al., 2021 (35) | South Nepal (8 districts) | General Health | Interview | Most respondents stated that the strongest impact was on the health services of immunization, maternal and childcare, and access to medicines | Difficulties with transportation to the hospital during lockdown and the need for private services that were often beyond financial resources. | Decreased compared to pre-pandemic period | | 21-75 | | Patients with chronic diseases, with fever did not receive medical care | |
| D’Ascenzo et al., 2021 (36) | Italy | Cardiology | Retrospective | Cardiology department admissions increase during pandemic | Increased mortality rate | Increased compered to pre-pandemic (cardiac patients only) | | 65+ | | Three periods were compared: during the pandemic peak, the period shortly before the pandemic outbreak in 2020, and separately in 2019 | |
| Chu et al.,2021 (37) | Canada (Ontario) | Telemedicine | Retrospective | Increase in online visits during COVID-19 pandemic | Increase in online visits regardless of age but mainly in patients over 65 years old | | Increased compered to pre-pandemicperiod | | 0-79 | | Study period: 1.01.2012, to 30.06. 2020; Study group: patients living in urban and rural areas. |
| Opinc et al., 2021 (38) | Poland | Rheumatology | Questionnaire | 77.57% of respondents experienced a restriction to healthcare services during the COVID-19 pandemic; 56.06% of respondents had a specialist clinic appointment cancelled; 31.77% stated that they did not receive medical care when needed. | 16.04% of respondents noted improvement in the quality of telemedicine services | Decreased compared to pre-pandemic period | | Mean age 41.52 | | Study group: 107 respondents; 90.65% were women | |
| Gómez - Ramiro et al., 2021 (39) | Spain | Mental Health | Retrospective | A significant overall decrease by 37.9% in psychiatric emergency admissions from pre-lockdown to on-lockdown period | Anxiety disorders were the most represented diagnoses both before and during lockdown | Decreased compared to pre-pandemic period | | Mean age 40.84 - 41.78 | | Comparison of pre-lockdown period (14.12.2019 to 13.03.2020) to on-lockdown (14.03.2020 to 12.06.2020) | |
| Ambrosetti et al., 2020 (40) | Switzerland | Mental Health - Emergency Department | Retrospective | Decrease of 17.5% in psychiatric admissions to the ED during COVID-19 | Patients admitted to the psychiatric ED were more frequently singles, were less likely self-referred, arrived more often with ambulance, more frequently overnight. | Decreased compared to pre-pandemic period | | - | | Comparison of two periods: 1.04.2020 to 15.05.2020, and 1.04. 2016 to 14.05.  2016 | |
| Zielasek et al., 2021 (41) | Germany | Mental Health | Retrospective | Decrease of 25% in psychiatric admissions during COVID-19 | The most pronounced decreases noted in intellectual disability- reduction of 51%; neurotic, stress-related, and somatoform disorders − 35%; affective disorders−34%; disorders of adult personality and behavior: −31% | Decreased compared to pre-pandemic period | | - | | Comparison of the daily admission numbers in the reporting periods of the years 2019 and 2020. | |
| Raffaldi et al., 2021 (42) | Italy | Pediatrics | Retrospective | Decreased in number of hospital admissions -70.48% | Higher reduction was reported in hospitals without Pediatric Intensive Care Unit (PICU) (-73·38%) than in those with PICU (-64·08%) Admissions with low priority decreased more than critical ones (-82·77% vs. 44·17% respectively) | Decreased compared to pre-pandemic period | | < 18 | | Comparison between the period 9.03.2020 to 3.05.2020, and the same period in 2019. | |
| Cagetti et al., 2021 (43) | Italy | Dental Health Services | Retrospective | Decrease in admissions during first and second lockdown −67.37% and −40.35% | Decrease −70.62% in Endodontic emergencies during the lockdown Traumas decreased during first lockdown −83.33% and the second −75.00%. Increase during the reopening period in other oral diagnosis +69.39% | Decreased compared to pre-pandemic period during lockdowns, increased in reopening period. | | ≤18 - >65 | | Comparison of four different periods of two weeks each: the pre-COVID period; the first lockdown; the reopening; the second lockdown | |
| Barasa et al., 2021(44) | Kenya | General Health | Retrospective | The outpatient utilization rate reduced; The bed occupancy rate significantly declined by 24.67%; Increase in health facility baby deliveries and measles vaccination | The health system experienced service delivery disruptions -malaria programme, notifications for TB, HPV vaccination; | Mostly decreased compared to pre-pandemic period | | - | | Comparison of periods between January 2019 and November 2020.March 2020 as the event month since it was the month where the first COVID-19 cases were first reported | |
| Cucu et al., 2021(45) | Romania | Mental Health/ General Health | Retrospective | The rate of admission due to mental and behavioural disorders decreased by: 55% for continuous hospitalization and 66-69% for day hospitalization | The rate of continuous hospitalization decreased due to: cardiovascular diseases by 46.99%, digestive diseases by 47.37%, for respiratory diseases by 37.28% | Decreased compared to pre-pandemic period | | - | | Comparison of periods between January and December 2018, between January and December 2019, and between January and December 2020 | |
| Doubova et al., 2021(46) | Mexico | General Health | Retrospective | Decrease in: sick child visits and contraceptive services by approx. 50%, the number of diabetes, hypertension and antenatal care consultations by approx. 33,3%, vaccinations by 36% and the number of deliveries attended at IMSS by 10% | Most affected service utilisation was breast and cervical cancer screening declining by 79% and 68%. | Decreased compared to pre-pandemic period | | - | | Comparison between the pre-COVID-19 period (January 2019 to March 2020) and the COVID-19 period (April to December 2020) | |
| Nguyen et al. 2021(47) | Bangladesh | Antenatal, maternal and child healthcare | Questionnaire | Decrease in antenatal care services , anthropometric measurements for women and children, prescription of and counselling on iron and folic-acid (IFA) and calcium supplementation. | Decrease in services provision and utilasation | Decreased compared to pre-pandemic period | | Average age for: pregnant women - 23 years, mothers - 25 years, children - 10 months. | | Comparison between the pre-pandemic period (February 2020), lockdown (March–May 2020) and pandemic period ( September–October 2020) | |
| Siedner et al., 2021(48) | South Africa | General Health | Retrospective | The small reduction in clinic visits for adult patients;  Decrease by 60% in child health visits for children between 1-5 years old; | No change in in clinic visitation for chronic non-communicable diseases and HIV-related clinical visits for adults; | Mostly no change. Decrease only in child health visits. | | 0-46+ | | Comparison between four periods: the prelockdown period startin, the lockdown period from 28 March through 30 April 2020, the lockdown from 1 May through 31 May and the lockdown 1 June through our data abstraction date (30 June) | |
